# Difference in glycerol levels between leukemia and normal bone marrow stem cells

**DOI:** 10.3892/ol.2014.2142

**Published:** 2014-05-13

**Authors:** YING-SONG QIN, DAN-XIA BU, YING-YING CUI, XIANG-YU ZHANG

**Affiliations:** 1 Department of Pathophysiology, Xuzhou Medical College, Xuzhou, Jiangsu 221002, P.R. China; 2 Department of Pathology, Affiliated Hospital of Xuzhou Medical College, Xuzhou, Jiangsu 221002, P.R. China; 3 State Key Laboratory of Oncogenes and Related Genes, Shanghai Cancer Institute, Renji Hospital, School of Medicine, Shanghai Jiao Tong University, Shanghai 200032, P.R. China

**Keywords:** leukemia, stem cells, glycerin, AQP9

## Abstract

Aquaglyceroporin 9 (AQP9) is considered to be involved in numerous types of carcinogenic processes, particularly in liver carcinoma. AQP9 expression is significantly decreased in the human hepatocellular carcinoma when compared with the non-tumourigenic liver, which leads to increased resistance to apoptosis. In addition, AQP9 is permeable to glycerol and urea. The involvement of AQP9 in leukemia has not been fully delineated. It is proposed that abnormal proliferation of hematopoietic stem cells (HSCs) contributes to leukemia carcinogenesis. Therefore, the present study aimed to investigate the possible roles of AQP9 in HSCs and its effect on the intracellular glycerol content. HSCs and non-HSCs (nHSCs) were isolated via magnetic-activated cell sorting and then subjected to flow cytometry for evaluation of purity. White blood cells (WBCs) were isolated from peripheral blood from healthy volunteers. Furthermore, AQP9 expression was examined at the mRNA and protein levels using western blotting and reverse transcription-polymerase chain reaction (RT-PCR). The glycerol content of HSCs, nHSCs and WBCs was evaluated by ELISA. Finally, in order to observe the morphology of HSCs and nHSCs, a blood smear was conducted and the cells were observed with Wright-Giemsa staining. The results indicated that the glycerol content in the HSCs was markedly greater than that in the nHSCs. AQP9 mRNA and protein expression was not detected in the HSCs and nHSCs, but was identified in the WBCs. Moreover, the HSC morphological characteristics included round or oval cells with round, slightly oval or irregularly shaped nuclei. Additionally, the nuclei occupied almost the entire cell, were located in the middle or were biased toward one side, and were stained light purple or red. Overall, our results indicated that intracellular glycerol is involved in HSC proliferation, despite the fact that glycerol is not mediated by AQP9. Hence, our findings may be useful in further understanding the mechanism of leukemia carcinogenesis, and these data may be valuable in developing future therapeutic strategies.

## Introduction

Leukemia is one of the leading causes of death among patients suffering from malignant hematological disorders (1). Despite the fact that great advances have been made in the treatment of leukemia, the overall survival rate has not greatly elevated (1,2). Hence, the understanding of the mechanism by which neoplastic cells proliferate in leukemia is of great importance.

It is known that alternation between the processes of dormancy and proliferation is very common in the human body, such as during wound healing, liver tissue regeneration and inflammatory proliferation (3–5). Moreover, mucosal epithelium in the respiratory tract, the digestive tract and the genital ducts is in a dynamic division process (6). Initiation of the abovementioned processes is based on the proliferation of original cells that possess stem cell properties. However, all of the aforementioned processes must occur under precise control, otherwise neoplasms may occur.

Abnormal alternation of hematopoietic stem cells (HSCs) is involved in the pathogenesis of leukemia. HSCs are the precursors of all blood cells. They maintain the dynamic balance between self-renewal and multipotential differentiation following the formation of the hematopoietic stem cell pool. Hematopoiesis is a complicated process that involves the interaction among hematopoietic stem/progenitor cells, the hematopoietic microenvironment and hematopoietic growth factors. It satisfies the requirement for the renewal and replacement of 1,012 blood cells from at least eight lines each day. Therefore, metabolism of numerous blood cells may be sustained by a minor subset of HSCs (7).

In nature, seed germination is also a process that alternates between dormancy and proliferation (8). The common characteristic of seeds is that oil is found in the cotyledon. In addition to providing nutrients, the oil likely maintains the dormant state of the seeds. The proliferation process begins when seeds incur water. Hence, we hypothesize that the rehydration process may be the key point in the initiation of cell proliferation. Therefore, in the human body, there may be a process that is analogous to the oil/water exchange that occurs in plants.

Aquaglyceroporin 9 (AQP9) was first discovered in adipose tissue, then in leukocytes (9), the liver (10), testicle (10), spleen (10)and brain (10). It is the only known aquaglyceroporin that expressed in the hematopoietic system (11). AQP9 is permeable to a number of small molecules, such as water and glycerol (13). High viscosity of the concentrated glycerol may slow down the biochemical processes in the cells and its water-soluble properties facilitate the replacement of water with glycerol. Therefore, we speculated that the difference in AQP9 expression between the quiescent normal HSCs and the proliferating malignant HSCs, and the difference in intracellular glycerol content between these two types of HSCs, may be involved in the mechanisms of hematopoietic tumorigenesis. The aim of the present study was to investigate the AQP9 expression in normal and leukemic HSCs, and to identify the possible effects of AQP9 in leukemia progression.

## Materials and methods

### Bone marrow and peripheral blood specimens

For this study, bone marrow specimens were obtained from six patients with leukemia and from thoracotomy patients without hematology diseases and pathogen infection, respectively, at the Hematology Department of the Affiliated Hospital of Xuzhou Medical College (Xuzhou, China). The peripheral blood was obtained from three healthy volunteers, who were students at Xuzhou Medical College, and reverse transcription-polymerase chain reaction (RT-PCR) and western blot analysis were performed to indicate the white blood cell (WBC) groups.

Following isolation of the mononuclear cells from each bone marrow sample using Ficoll-Hypaque screening (Shanghai Biochemical Co., Ltd., Shanghai, China), the hematopoietic stem cells were separated out by magnetic-activated cell sorting (MACS). The study was approved by the ethics committee of the Affiliated Hospital of Xuzhou Medical College, and all patients and healthy volunteers provided informed consent.

### MACS

StemSep^®^ Human Primitive Hematopoietic Progenitor Cell Enrichment kit (14057) and EasySep^®^ Magnetic Nanoparticles (19150.1) were purchased from Hangzhou Baitong Biotech Co., Ltd. (Hangzhou, China). Cells were labeled with primary monoclonal mouse anti-human CD34/38 antibody, magnetically labeled with rabbit anti-mouse microbeads and separated on MACS column (all Hangzhou Baitong Biotech Co., Ltd.). All the procedures were carried out according to manufacturer’s instructions. Cells were then positively enriched on micro beads.

### Purity of the sorted cells measured by flow cytometry

The final concentrations of the sorted stem cells and non-stem cells were adjusted to 1×10^6^ cells/ml, then both mouse anti-human CD38 FITC monoclonal antibody (eBioscience, Inc., San Diego, CA, USA) and mouse anti-human CD34 PE monoclonal antibody (eBioscience, Inc.) were added at a concentration of 5 μl/ml. Following incubation on ice in the dark for 30 min, the purity rate was analyzed on a FACScan flow cytometer (Becton Dickinson, San Jose, CA, USA). The ratio of sorted stem cells versus bone marrow cells as well as peripheral blood mononuclear cells was determined using Trypan blue staining (Gibco-BRL, Eggenstein, Germany).

### Western blot analysis

Cells from the from L-HSC, L-nHSC, N-HSC, N-nHSC and WBC groups were collected and lysed with 200 μl lysate (Beyotime Institute of Biotechnology, Haimen, China), then centrifuged to pellet the cell debris. Proteins were separated on SDS-PAGE gels (Shanghai Shenggong Biotechnological Ltd., Shanghai, China) and transferred to polyvinylidene difluoride (PVDF) membranes (Millipore, Billerica, MA, USA). Following this, the PVDF membranes were rinsed using washer buffer (phosphate-buffered saline with Tween 20; Zhongshan Golden Bridge Biotech Co., Ltd., Beijing, China), and then were blocked with 5% non-fat milk. Immunoblotting was performed with primary mouse monoclonal antibodies specific for AQP9 and β-actin (Santa Cruz Biotechnology, Inc., Santa Cruz, CA, USA), followed by secondary monoclonal rabbit anti-mouse alkaline phosphatase-conjugated antibody (Zhongshan Golden Bridge Biotech Co., Ltd.). The proteins were detected with NBT/BCIP chemiluminescence reagent (Promega, Madison, WI, USA). Densitometric intensity was measured with the Image-J microscopy image analysis system (Shanghai Furi Science and Technology Co., Ltd., Shanghai, China) and normalized against a β-actin internal control.

### Detection of AQP-9 mRNA expression by RT-PCR

Total RNA was isolated from the different groups of cells using the RNAprep pure kit (Tiangen Biotech Co., Ltd., Tianjin, China), and the purity and concentration of RNA was determined by the NanoDrop 1000 spectrophotometer (Thermo Fisher Scientific, Inc., Waltham, MA, USA). The cDNA was then prepared from 5 μg of total RNA by reverse transcription using TianScript cDNA First Strand cDNA Synthesis kit (Tiangen Biotech Co., Ltd.), and 2 μl of the cDNA was amplified for 40 cycles with specific primers for AQP1, AQP9 and GAPDH (Tiangen Biotech Co., Ltd.) (Table I). PCR reactions were initiated with incubation at 94°C for 3 min, followed by 38 cycles of 94°C for 30 sec, 58°C for 30 sec and 72°C for 1 min. Reactions were completed with a 72°C, 5 min extension. Subsequently, the targeted DNA was confirmed by agarose-gel electrophoresis, the intensity of each band was determined by a gel digital image analysis system (Furi FR-980; Shanghai Furi Science and Technology Co., Ltd.) and normalized against a GAPDH internal control.

### Detection of the glycerol contents of each group

The cell glycerol content was measured in all experimental groups using the BG Glycerin ELISA kit (Shanghai Lanji Biotech Co., Ltd., Shanghai, China). The cell concentration was adjusted to 8×10^4^ cells/ml; cells were lysed by repeatedly freezing and thawing; and 50 μl of standard, sample or distilled water was added to each well of a 96-well microtiter plate, according to the manufacturer’s instructions. A standardized preparation of the polyclonal mouse anti-human horseradish peroxidase (HRP)-conjugated antibody (Shanghai Lanji Biotech Co., Ltd.) specific for glycerin was added to each well to bind the glycerol immobilized on the plate, then the HRP-linked solution was added. After incubating for 1 h at 37°C, the plate was washed thoroughly to remove all unbound components. Then, substrate solutions A and B were added to each well. The enzyme (HRP) and substrate were allowed to react over a short period (5 min). The enzyme-substrate reaction was terminated by the addition of a sulfuric acid solution and the color change was measured spectrophotometrically at a wavelength of 450 nm.

### Smear of leukemia and normal bone marrow cells and Wright-Giemsa staining

The two types of bone marrow were processed routinely for smearing on slides coated with polylysine. After being dried, the samples were stained with Wright-Giemsa dye solution (Nanjing Jiancheng Bioengineering Institute, Nanjing, China) in order to observe the cellular characteristics. An oil microscope (Olympus BX51; Olympus, Tokyo, Japan) was used to carefully observe the size and shape of the hematopoietic stem cells, to determine whether the hematopoietic stem cells could be distinguished from their morphology alone.

### Statistical analysis

All data were analyzed with SPSS statistical software (version 13.0; SPPS, Inc., Chicago, IL, USA). Results are expressed as the mean ± standard deviation. Multiple comparisons were assessed by one-way analysis of variance, and analysis of differences between groups was carried out using Student-Newman-Keuls analysis. P<0.05 was considered to indicate a statistically significant difference.

## Results

### Effective isolation of stem cells from bone marrow

**Figure 1:**
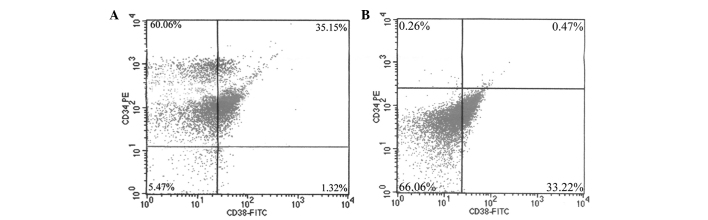
FACS analysis of CD34 and CD38 antigen expression in (A) bone marrow stem cells and (B) bone marrow non-stem cells. The cells were stained with mouse anti-human CD34 PE monoclonal antibody for detection of CD34 antigen expression, and with mouse anti-human CD38 FITC monoclonal antibody for detection CD38 antigen expression.

Stem cells and non-stem cells were isolated from the bone marrow by MACS, labeled with mouse anti-human CD34 PE and CD38 FITC antibodies, and analyzed by FACS. With regard to stem cells, the purity rate of the sorted CD34^+^/CD38^−^, CD34^+^/CD38^+^, CD34^−^/CD38^−^ and CD34^−^/CD38^+^ cells was 60.06, 35.15, 5.47 and 1.32%, respectively (Fig. 1A). Additionally, with regard to non-stem cells, the purity rate of the sorted CD34^−^/CD38^−^ CD34^−^/CD38^+^ CD34^+^/CD38^−^ and CD34^+^/CD38^+^ cells was 66.06, 33.22, 0.26 and 0.47%, respectively (Fig. 1B).

The ratio of leukemia bone marrow stem cells versus bone marrow cells and mononuclear cells was ~3 and ~17.3%, respectively. While the ratio of normal bone marrow stem cells versus bone marrow cells and mononuclear cells was ~0.8 and ~8%, respectively (Fig. 1).

### AQP9 protein expression is not observed in bone marrow stem cells or non-stem cells

**Figure 2:**
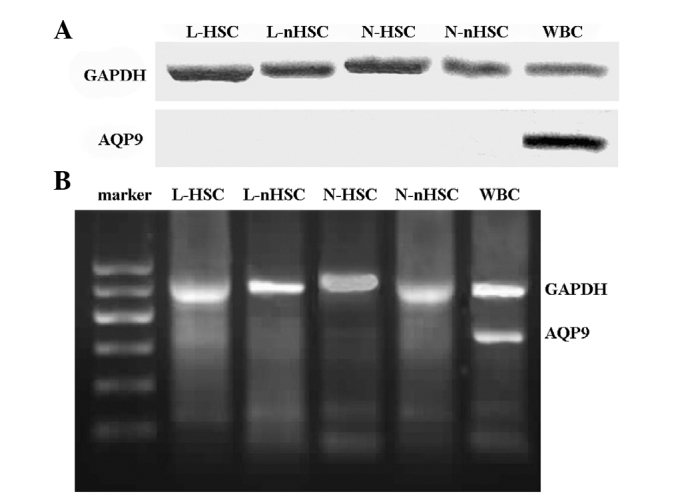
AQP9 expression at the (A) protein and (B) mRNA levels in L-HSCs, L-nHSCs, N-HSCs, N-nHSCs and WBCs. AQP9 expression was only detected in the WBCs. L-HSCs, hematopoietic stem cells (HSCs) isolated from leukemia patients; L-nHSCs, non-HSCs (nHSCs) isolated from leukemia patients; N-HSCs, HSCs isolated from thoracotomy patients; N-nHSCs, nHSCs isolated from thoracotomy patients; WBCs, white blood cells.

The expression of AQP9 protein was only detected in the peripheral blood (in the WBCs) (Fig. 2A). The results confirmed that AQP9 protein was not expressed in the remainder of the experimental groups.

### AQP9 mRNA expression in peripheral blood WBCs

The expression of AQP9 mRNA was only detected in peripheral blood (in the WBCs) (Fig. 2B); AQP9 mRNA expression was not detected in the remainder of the experimental groups.

### Glycerol downregulation in non-leukemia hematopoietic stem cells

**Figure 3:**
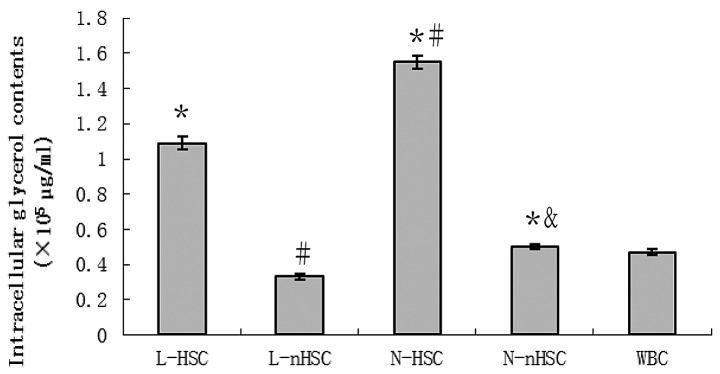
Intracellular glycerol contents in L-HSCs, L-nHSCs, N-HSCs, N-nHSCs and WBCs. The N-HSCs exhibited the greatest concentration of glycerol among the five groups, and the glycerol content of the L-HSCs was less than that of the N-HSCs, but greater than that of the remaining three groups. ^*^P<0.05 vs. WBC; ^#^P<0.05 vs. L-HSC; ^&^P<0.05 vs. N-HSC. L-HSCs, hematopoietic stem cells (HSCs) isolated from leukemia patients; L-nHSCs, non-HSCs (nHSCs) isolated from leukemia patients; N-HSCs, HSCs isolated from thoracotomy patients; N-nHSCs, nHSCs isolated from thoracotomy patients; WBCs, white blood cells.

The glycerol content in all experimental groups is shown in Fig. 3. The glycerol concentration was identified to be significantly higher in the N-HSCs compared with the N-nHSCs, in the N-nHSC compared with the L-nHSCs and in the L-HSCs compared with the L-nHSCs (P<0.05 for all). The concentration of glycerol in the L-HSCs and N-HSCs was significantly higher than that in the WBCs (P<0.05 for both), and that in the L-nHSCs was significantly lower compared with that in the WBCs (P<0.05).

### Bone marrow and stem cell smear

**Figure 4:**
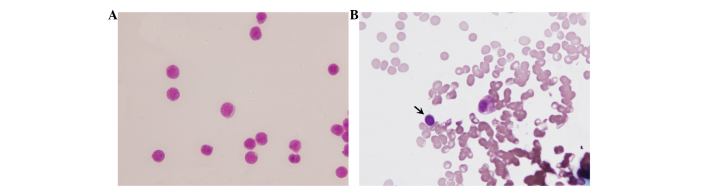
Morphology of (A) normal and (B) leukemia bone marrow cells. The arrow indicates a hematopoietic stem cell that is irregular in shape.

The morphology of stem cells of normal bone marrow is typical, while that of the leukemia stem cell is atypical. Under the oil microscope, primitive cells of the same shape and size were observed in both the bone marrow and stem cell smears. These primitive cells were round or oval, and the nuclei were round, slightly oval or irregular in shape. In addition, the nuclei occupied almost the entire cell, were located in the middle or were biased toward one side, and were stained light purple or red. The chromatin was tender, uniformly distributed and deeply stained, without any phenomenon of aggregation. The nuclear membrane was neat and thin, and the nucleus had three to six nucleoli. The cytoplasm was seldom and without visible particles, and was stained light blue (Fig. 4).

## Discussion

AQP9 is widely expressed in a number of tissues which are permeable to water, glycerol and urea. AQP9 is also involved in the glycerol uptake by hepatocyte for gluconeogenesis (14). Certain studies have found that AQP9 is abundant in peripheral blood leukocytes, which is consistent with the results of the current study (9). When the leukemia K562 cell line was transfected with human AQP9 cDNA, trisenox uptake was found to increase (11), which may lead to leukemia cell death (15). Therefore, the downregulation of AQP9 may present as a target for cancer therapy (16). Qu *et al* (17) verified via *in situ* hybridization that AQP9 is expressed in cytotrophoblasts and syncytiotrophoblasts of the placental epithelial cells of the amnion in healthy pregnant women. It is speculated that AQP9 is associated with maternal-fetal water and solution exchange. Damiano *et al* (18) verified by histochemistry and western blotting that the expression of AQP9 was elevated in the preeclampsia placenta; however, the functional ability of AQP9 for water permeability was decreased, suggesting that AQP9 plays an important role in pre-eclampsia. Badaut *et al* (19) suggested that AQP9 can regulate the metabolic balance between brain parenchyma and cerebrospinal fluid. Yamamoto *et al* (20) showed that cerebral edema, which is due to cerebral ischemia and hypoxia, is closely associated with AQP9; AQP9 functions in maintaining the internal environment homeostasis and in lactate buffering. As a member of the aquaglyceroporin family, AQP9 is abundantly expressed on the sinusoid surface of hepatocytes, indicating that AQP9 is involved in the liver-blood glycerol exchange (21). Additionally, certain studies have suggested that AQP9 is involved in hepatocyte lipid metabolism and steatosis (22–24). Apart from its contribution to neoplasm pathogenesis, AQP9 has also been implicated in the process of inflammation; a previous study indicated that a strong increase in AQP9 transcripts was observed in synovial tissues from patients with osteoarthritis and rheumatoid arthritis (25).

The present study indicated that glycerol was present in the cells of all five groups, and that the glycerol content in the L-HSCs was significantly lower than that in the N-HSCs (P<0.05). Similarly, the concentration of glycerol in the L-nHSCs was significantly lower compared with that in the N-nHSCs (P<0.05). Moreover, the glycerol content in the L-HSCs was significantly higher than that in the L-nHSCs (P<0.05), and the same trend was observed between the N-HSCs and the N-nHSCs (P<0.05). Since the proliferation rate of L-HSCs is markedly higher than that of N-HSCs, and that of L-nHSCs is markedly higher than that of N-nHSCs, these results indicated that the cell proliferation state maybe associated with the intracellular glycerol content; the higher the intracellular glycerol content, the lower the proliferation rate. This is a novel insight into the mechanism underlying leukemia cell proliferation, which may be useful for the growth intervention of leukemia cells, as altering the intracellular glycerol content may be beneficial for decreasing the tumor cell proliferation rate.

However, our results also showed that no AQP9 expression was detected in the L-HSCs, L-nHSCs, N-HSCs or N-nHSCs; while AQP9 was identified to be expressed in the WBCs of the normal peripheral blood. Although AQP9 is the only known aquaglyceroporin that is expressed in the hematopoietic system, there may be other pathway for the glycerol to enter these normal or abnormal bone marrow hematopoietic cells. The possible mechanism for the cell growth inhibition induced by an increase in the intracellular glycerol concentration may lead to an increase in the viscosity of the cytoplasmic fluid, which may subsequently lead to the decelerated movement of signal molecules. As a result, the cell growth is inhibited. The concrete mechanisms are required to be further investigated.

Glycerol is the carbon backbone of triacylglycerol and represents an important metabolite for the control of fat accumulation and glucose homeostasis; glycerol serves as the major substrate for hepatic gluconeogenesis during periods of fasting (26). It has been demonstrated that glucose metabolism is altered in neoplastic cells; the glycolytic rate is much higher than that in normal cells. Aerobic glycolysis is a hallmark of cancer, and cancer cells become highly glycolytic (27). Hence, we speculate that the influx of glycerol into leukemia stem cells or normal bone marrow stem cells is markedly greater than that of non-stem leukemia cells or non-stem normal bone marrow cells; however, the increased glycerol is immediately metabolized for producing glucose, and thus the cells enter into aerobic glycolysis to produce sufficient energy. Previously, Sun *et al* (27) indicated that pyruvate kinase isoenzyme type M2 is involved in this process. It is therefore possible that the glycerol content is merely a reflection of the active glucose metabolism of leukemia stem cells.

In conclusion, the present study identified that the glycerol content of leukemia stem cells was significantly lower than that of normal bone marrow stem cells, while both cell types were deficient of AQP9. The glycerol content is reduced in leukemia stem cells compared with that in normal bone marrow stem cells. The excessive aerobic glycolysis is not only attributable to glycerol, but also to other substances, such as amino acids. It is possible that amino acids dominate glycerol in this aerobic glycolysis for leukemia stem cells. This study provides a novel insight into understanding leukemia tumorigenesis, which may be utilized for leukemia prevention.

## Figures and Tables

**Table I:** Specific primers for AQP9 and GAPDH.

Primer	Sequence	Length
H-AQP9-P1	5-GAGCAGCTTAGCGAAAG-3	344 bp
H-AQP9-P2	5- CACCAGCAAAGGACATA-3	
H-GAPDH-P1	5-AGGTCGGAGTCAACGGATTTG-3	532 bp
H-GAPDH-P2	5-GTGATGGCATGGACTGTGGT-3	
